# 新型3D色谱固定相结合气相色谱-质谱法测定染发剂中多种限用和禁用成分

**DOI:** 10.3724/SP.J.1123.2023.11018

**Published:** 2024-10-08

**Authors:** Peng ZHANG, Kuan LI, Jin ZHOU, Weina HAN, Yongrui HE

**Affiliations:** 山东第二医科大学药学院, 山东 潍坊 261053; School of Pharmacy, Shandong Second Medical University, Weifang 261053, China

**Keywords:** 气相色谱-质谱法, 3D固定相, 三蝶烯, 染发剂, gas chromatography-mass spectrometry (GC-MS), 3D stationary phase, triptycene (TP), hair dyes

## Abstract

染发剂(hair dyes, HDs)主要成分为多种苯系物、胺类和酚类化合物,具有致敏性、致畸变性和致癌性等危害,近年来受到广泛关注。目前传统的分析检测方法和商品化色谱柱大多存在定性难度大或定量不准确等问题,亟需开发分析检测新技术和新型固定相。同时,染发剂成分复杂、基质干扰较大,需要合适的样品前处理方法。本研究基于3D非极性刚性结构单元三蝶烯(TP),结合极性柔性链聚乙二醇(PEG),设计、合成了蝶烯衍生物TP-PEG,作为色谱柱固定相。采用气相色谱-质谱法(GC-MS)定量分析了22种染发剂成分,并对4种实际样品进行了检测分析。该固定相由非极性3D刚性结构和柔性链状极性结构两部分构成,具有“刚柔并济”的结构特点,扩大了对非极性和极性分析物的选择范围。实验结果表明,不同极性的分析物在该固定相上均展现了尖锐、对称的峰形,且22种分析物在该色谱柱上都达到了基线分离,为染发剂各成分的检测分析提供了保障。结果表明,22种染发剂成分在各自范围内线性关系良好,相关系数均≥0.9985。各染发剂成分在3个添加水平下的平均回收率为89.2%~103.2%, RSD均小于5%。与传统方法相比,该方法效率高,准确性好,验证了新型固定相优异的分离性能以及建立的GC-MS检测方法的有效性。

## Abstract (en)

Hair dyes (HDs) are mainly composed of various benzene series, amines, and phenolic compounds. These ingredients are well known to have allergenic, teratogenic, and carcinogenic properties. As such, the presence of these ingredients in HDs has received increased attention in recent years. At present, the applications of traditional analytical and detection methods and commercial chromatographic columns are limited by problems such as poor qualitative analysis and inaccurate quantification. Thus, the development of new analytical and detection technologies and stationary phases is an urgent endeavor. Moreover, HDs contain complex compounds and exhibit significant matrix interference. Hence, appropriate sample pretreatment methods are necessary to analyze HDs. In this study, the 3D nonpolar rigid structure of triptycene (TP) was combined with the polar flexible chains of polyethylene glycol (PEG) to design and synthesize a TP derivative, TP-PEG, as a stationary phase for chromatographic columns. The stationary phase enabled the expansion of the selection range for polar and nonpolar analytes. Subsequently, gas chromatography-mass spectrometry (GC-MS) was used to quantitatively analyze 22 ingredients in HDs. The experimental results demonstrated that analytes with different polarities exhibited sharp and symmetrical peak shapes on the stationary phase, and all 22 analytes achieved baseline separation on the chromatographic column. The 22 ingredients in HDs showed good linear relationships within their respective ranges, with correlation coefficients greater than 0.9985. The average recovery rates at three spiked levels were in the range of 89.2%-103.2%, and RSDs were less than 5%. Compared with traditional methods, the proposed method has higher efficiency and better accuracy, thus verifying the excellent separation performance of the new stationary phase and the effectiveness of the established GC-MS detection method. The findings indicated the applicability of the developed method to the detection and analysis of various compounds in HDs.

在过去的几十年中,化妆品市场规模不断扩张,中国已成为全球第二大化妆品消费市场,增速超全球平均速度,预计到2050年中国将成为全世界最大的化妆品消费市场。在庞大的化妆品市场中,染发剂(hair dyes, HDs)的使用占比很大,其有害成分带来的影响逐渐显露^[1,2]^。HDs主要成分为各类苯系物、胺类及酚类化合物,这些化合物中一些异构体或衍生物具有致敏性、致突变性,部分可引起重金属离子蓄积中毒、皮肤色素沉积、接触性皮炎、手指溃疡等^[3]^。HDs有害成分可能会诱发膀胱癌、乳腺癌、淋巴癌等癌症的发生^[4]^。根据相关部门调查显示,染发剂的大量使用也会带来空气中苯系物含量超标,对空气质量造成负面影响^[3]^。因此,对染发剂中苯系物、胺类和酚类化合物的含量测定至关重要。

目前常见的HDs成分的分析方法有气相色谱-质谱法(GC-MS)^[5⇓-7]^、高效液相色谱-质谱法(HPLC-MS)^[8⇓-10]^、电化学方法等^[11⇓-13]^。其中,GC-MS由于检测速度快、灵敏度高常被用于工业、食品、环境、医药、化妆品等领域的分离分析^[14,15]^。GC作为一种成熟的分离、分析技术,在实现有效分离的过程中主要依靠色谱固定相。因此,固定相的研究和开发是影响气相色谱发展的重要因素之一^[14⇓⇓-17]^。值得注意的是,染发剂中常见成分胺类及酚类化合物或其异构体具有较强的活性,在固定相上吸附作用较强、易拖尾,实现胺类或酚类异构体的基线分离具有一定的挑战性,且针对胺类和酚类化合物的固定相或商品柱较少^[5]^。因此,新型固定相材料用于GC-MS分离复杂分析物非常必要。合适的固定相和GC较高的灵敏度在难分离化合物或微量成分的检测中起着至关重要的作用。同时,复杂样品检测过程中易受到杂质的影响,使其难以定性或存在定量不准确的问题,采取合适的萃取前处理技术可有效降低复杂样品中杂质成分的干扰^[18,19]^。

三蝶烯(triptycene, TP)由3个苯环通过9,10位桥头堡的碳原子相互铰链而成,具有3D刚性对称结构,其衍生物被广泛运用于光电材料、聚合物气体吸附膜分离、储气、分子识别和超分子组装等领域^[20,21]^。聚乙二醇(polyethylene glycol, PEG)是最常用的GC固定相之一,常被用作商品柱固定相材料,如HP-INNOWAX柱。本工作基于3D非极性刚性结构的三蝶烯,结合极性柔性链PEG,设计、合成了蝶烯衍生物(triptycene-polyethylene glycol, TP-PEG)。该固定相结构含有非极性和极性两部分,对不同极性的分析物均可表现出较强的分子间作用力^[20⇓-22]^。同时,高效、快速的样品前处理技术不仅可以提高分析方法的检测灵敏度、准确度、选择性,还可以降低或避免杂质对仪器带来的污染和损害。目前,染发剂常用的萃取方法有超声辅助溶剂提取、液液萃取、液液微萃取、固相萃取等^[6,7]^。其中,超声辅助溶剂提取是染发剂样品最常用、最便捷的萃取方法,可有效降低样品中的基质干扰,提高检测的灵敏度和准确度。

本工作通过设计、合成固定相TP-PEG,将TP-PEG作为GC色谱柱固定相,同时采用超声辅助溶剂提取技术有效降低基质干扰,以染发剂中的多种限用和禁用成分为研究对象、采用GC-MS进行分离、分析,建立了一种同时分析22种染发剂成分的分析方法,解决了染发剂中部分成分存在检测难、检测不准确的问题。

## 1 实验部分

### 1.1 仪器、试剂与材料

Agilent 7890A-5975C气相色谱-质谱联用仪,配有火焰离子检测器(FID)、自动进样,Agilent科技(中国)有限公司产品;全自动加速溶剂萃取仪ASE300, Thermo公司产品;超声波清洗器KQ-300DB,昆山市超声仪器有限公司产品;pH计Delta320,梅特勒-托利多仪器(上海)有限公司产品;分析天平BSA224S-CW,德国赛多利斯公司产品;涡旋振荡仪Vortex2,德国IKA公司产品;离心机LG10-24A,北京雷勃尔离心机有限公司产品;0.22 μm滤膜,上海安谱实验科技股份有限公司产品。

乙醇:色谱纯,德国默克公司产品;2 g/L抗坏血酸:分析纯,国药集团化学试剂有限公司产品;22种染发剂标准品(色谱纯)均购于麦克林公司。

### 1.2 标准储备液的制备

标准储备液:称取各标准品均约50 mg(精确至0.1 mg),置于10 mL容量瓶中,加入乙醇-2 g/L抗坏血酸水溶液(1∶1, v/v)溶解,冰水浴超声,放置至室温后,加入乙醇-2 g/L抗坏血酸水溶液(1∶1, v/v)定容至10 mL,配制成5 mg/mL的染发剂成分单标准储备液。

标准工作液(现用现配):准确吸取各成分标准储备液于棕色容量瓶中,用乙醇-2 g/L抗坏血酸水溶液(1∶1, v/v)稀释,配制2.5~50 μg/mL的系列混合标准溶液。

### 1.3 样品前处理

称取样品约0.5 g(精确至0.1 mg)于25 mL具塞刻度管中,加入乙醇-2 g/L抗坏血酸水溶液(1∶1, v/v)15 mL,涡旋1 min,冰水浴超声提取20 min,放置至室温,用乙醇-2 g/L抗坏血酸水溶液(1∶1, v/v)定容至25 mL。取10 mL样品溶液于离心管中,加入10 mL正己烷,涡旋1 min,混匀,然后以4000 r/min离心5 min,弃去正己烷层,经0.22 μm微孔滤膜过滤,滤液作为待测溶液。

### 1.4 TP-PEG的合成

本工作使用的色谱柱为自制色谱柱,固定相TP-PEG的合成方法参考前期工作^[20]^。TP-PEG是由3D非极性结构和链状极性结构共同组成的一类具有两亲特性的三蝶烯材料(见图1)。以三蝶烯和聚乙二醇400为原料,对非极性部分的三蝶烯进行羟基化后得到三蝶烯-二羟基(TP-2OH),同时对极性部分PEG进行磺酰化,最后将非极性部分的TP-2OH与极性部分PEG连接得到最终产物,即为色谱固定相TP-PEG。

**图1:**
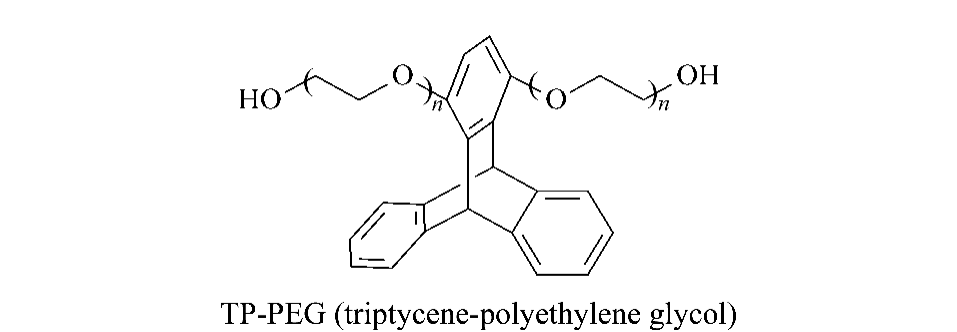
固定相TP-PEG的结构示意图

### 1.5 色谱条件

色谱柱:以TP-PEG为固定相,规格为30 m×0.25 mm的毛细管色谱柱;程序升温条件:50 ℃保持2 min,以5 ℃/min升温至230 ℃,保持50 min;载气(N_2_)流速1 mL/min,进样量0.6 μL;分流比20∶1。

质谱条件:电子轰击电离(EI)源;电子能量70 eV;传输线温度280 ℃;进样口温度230 ℃;四极杆温度150 ℃;溶剂延迟:5 min,采集方式为全扫描(SCAN)和选择离子监测(SIM)模式,全扫描范围:*m/z* 10~150。22种染发剂成分的保留时间、定性离子、定量离子等参数见表1。

**表1:** 22种染发剂成分的质谱参数

No.	Compound	Retention time/min	Primary ion (m/z)	Secondary ions (m/z)
1	phenol (苯酚)	6.41	94.46	66.05^*^, 39.42
2	N,N-diethyl p-diphenylamine (N,N-二乙基对二苯胺)	6.61	164.31	149.21^*^, 120.42
3	o-phenylene-diamine (邻苯二胺)	11.32	108.09	80.01, 53.13^*^
4	3,4-diaminotoluene (3,4-二氨基甲苯)	16.23	122.14	106.42^*^, 94.12
5	p-phenylene-diamine (对苯二胺)	17.21	108.72	80.21^*^, 53.02
6	2,5-diaminotoluene (2,5-二氨基甲苯)	19.16	122.07	106.51, 94.05^*^
7	2,6-diaminopyridine (2,6-二氨基吡啶)	19.61	109.19	81.43^*^, 53.11
8	2,4,5-trichlorophenol (2,4,5-trichlorophenol)	20.61	109.46	81.02^*^, 53.15
9	2-aminophenol (邻氨基苯酚)	21.83	195.13	124.45, 133.21^*^
10	4-methylaminophenol (4-甲氨基苯酚)	23.25	123.45	108.63^*^, 94.22
11	p-aminophenol (对氨基苯酚)	23.41	109.06	81.03^*^, 53.25
12	4-amino-m-cresol (4-氨基-间甲酚)	25.63	123.17	77.04, 95.21^*^
13	5-amino-o-cresol (5-氨基-邻甲酚)	26.61	123.34	78.08, 106.15^*^
14	m-aminophenol (间氨基苯酚)	26.68	109.02	81.12^*^, 53.03
15	2-amino-3-hydroxypyridine (2-氨基-3-羟基吡啶)	27.33	110.31	55.23^*^, 81, 03
16	4-nitro-o-phenylenediamine (4-硝基-邻苯二胺)	27.66	153.04	107.52^*^, 95.14
17	p-dihydroxybenzene (对苯二酚)	28.39	110.72	81.07^*^, 53.19
18	m-dihydroxybenzene (间苯二酚)	28.65	110.11	81.41^*^, 53.52
19	1,5-naphthalenediol (1,5-萘二酚)	32.12	160.05	131.21^*^, 103.12
20	2,7-naphthalenediol (2,7-萘二酚)	37.31	160.07	131.51^*^, 103.04
21	1-naphthol (1-萘酚)	37.75	144.11	116.23^*^, 89.02
22	2-naphthol (2-萘酚)	40.35	144.23	116.01^*^, 89.11

^*^ Quantitative ion.

## 2 结果与讨论

### 2.1 萃取溶剂的优化

染发剂主要由染料和基质组成,前处理净化的主要目的是减少基质干扰,提高染发剂成分的提取效率。本研究以有机溶剂-2 g/L抗坏血酸溶液(1∶1, v/v)作为萃取溶剂对样品进行前处理。实验对甲醇、乙醇、乙酸乙酯、乙醚、丙酮、三氯甲烷、正己烷7种有机溶剂进行了考察。结果表明,丙酮、三氯甲烷、正己烷3种有机溶剂无法完全溶解染发剂;乙醚在加入到样本中时发生化学反应,立即变成黑色;甲醇、乙醇与乙酸乙酯可以完全溶解染发剂并且没有变色。因此,实验进一步考察了甲醇、乙醇和乙酸乙酯作为有机溶剂时,22种染发剂成分的峰面积,如图2所示。结果表明,采用乙醇-2 g/L抗坏血酸水溶液(1∶1, v/v)萃取时,得到的染发剂成分峰面积最大。

**图2:**
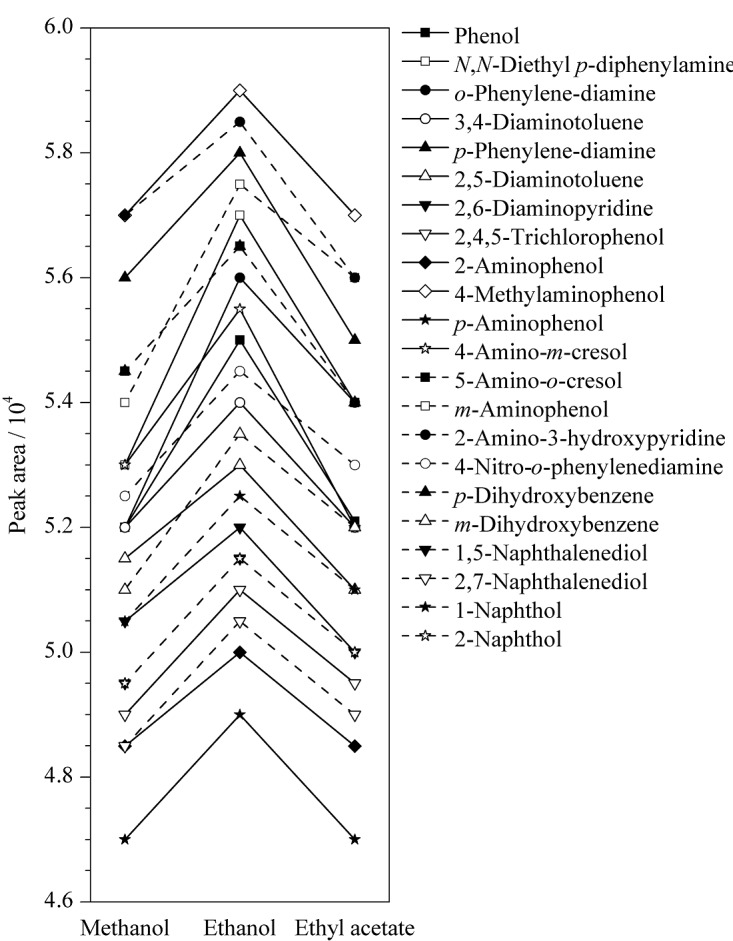
采用不同有机溶剂时22种染发剂成分的峰面积

### 2.2 超声时间的优化

本工作考察了超声时间(3、5、7 min)对分析物峰面积的影响,结果如图3所示。结果表明,随着超声时间的增加,分析物的峰面积先增加后降低。当超声时间为5 min时分析物的峰面积最大。因此,本工作选取超声振荡时间为5 min。

**图3:**
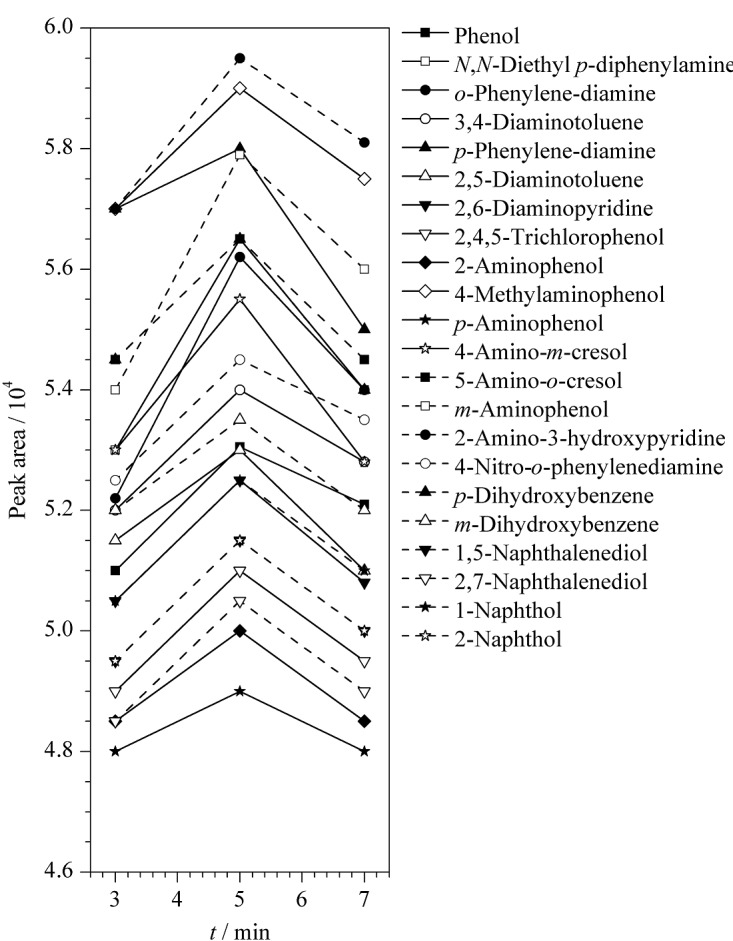
采用不同超声时间时22种染发剂成分的峰面积

### 2.3 质谱条件的优化

在电子轰击电离源下进行一级质谱全扫描,选择最佳强度的一级母离子;再对各成分进行二级质谱扫描,选择响应较强且无干扰的定性离子和定量离子;在SIM模式下进一步优化碰撞能参数。22种染发剂成分混合标准溶液的总离子流色谱图见图4,各化合物峰形对称且尖锐,完全能满足测试需求。

**图4:**
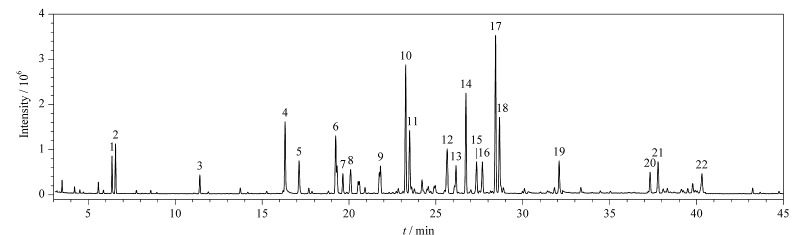
22种染发剂成分混合标准溶液的总离子流图

### 2.4 线性范围、检出限和定量限

配制系列混合标准溶液,在最佳实验条件下进行进样测定,以目标成分的峰面积(*Y*)对相应的质量浓度(*X*, mg/L)绘制标准工作曲线。在相应的范围内,22种染发剂成分线性关系良好,相关系数(*r*)均≥0.9985。通过空白基质加标试验,考察检出限(LOD)和定量限(LOQ),以信噪比(*S/N*)=3和10时的含量为LOD和LOQ,分别为15~35 μg/g和47~115 μg/g,结果见表2。

**表2:** 22种染发剂成分的线性方程、线性范围、相关系数、检出限和定量限

Compound	Linear equation	Linear range/(mg/L)	r	LOD/(μg/g)	LOQ/(μg/g)
Phenol	Y=40682X-91414	5-500	0.9989	15	53
N,N-Diethyl p-diphenylamine	Y=4693X-2192	5-500	0.9985	15	59
o-Phenylenediamine	Y=34032X-10404	5-500	0.9999	25	83
3,4-Diaminotoluene	Y=2756X+17.31	5-500	0.9999	20	64
p-Phenylenediamine	Y=3407X-60.29	5-500	0.9985	17	57
2,5-Diaminotoluene	Y=32467X-5902	5-500	0.9999	25	86
2,6-Diaminopyridine	Y=6873X-3593	5-500	0.9994	20	75
2,4,5-Trichlorophenol	Y=11870X-1564	5-500	0.9999	19	69
2-Aminophenol	Y=19379X-4868	5-500	0.9999	16	52
4-Methylamino-phenol	Y=12179X+4961	5-500	0.9993	15	57
p-Aminophenol	Y=8069X+517	5-500	0.9993	19	73
4-Amino-m-cresol	Y=16990X-3721	5-500	0.9999	25	87
5-Amino-o-cresol	Y=7918X-324	5-500	0.9996	35	115
m-Aminophenol	Y=9460X-1634	5-500	0.9999	30	101
2-Amino-3-hydroxypyridine	Y=19450X-15482	5-500	0.9992	25	79
4-Nitro-o-phenylenediamine	Y=12972X-3149	5-500	0.9999	19	61
p-Dihydroxybenzene	Y=9065X+688	5-500	0.9991	25	82
m-Dihydroxybenzene	Y=22318X-2107	5-500	0.9992	20	63
1,5-Naphthalenediol	Y=15165X-1369	5-500	0.9995	20	69
2,7-Naphthalenediol	Y=8431X-33012	5-500	0.9987	25	91
1-Naphthol	Y=10631X-39136	5-500	0.9989	15	47
2-Naphthol	Y=2661X-9289	5-500	0.9989	20	65

*Y*: peak area; *X*: mass concentration, mg/L.

### 2.5 回收率和精密度

在染发剂空白样品中分别添加3个水平(LOQ、5倍LOQ、10倍LOQ)的22种染发剂成分混合标准溶液进行加标回收试验,每个添加水平分别连续测定6次和连续测定3天,每天连续测定6次,分别计算各成分的回收率和日内、日间精密度(用峰面积的RSD表示)。结果表明,22种染发剂成分的平均加标回收率为89.2%~103.2%,回收率结果良好;RSD均小于5%,满足日内和日间精密度的要求。

### 2.6 实际样品的检测

采用本法测定了市售的4种染发剂样品(表3)。结果表明,不同品牌和颜色的染发剂中所含成分主要包括对苯二胺、邻氨基酚、对氨基酚、间氨基酚、对苯二酚及间苯二酚。

**表3:** 市售染发剂样品的分析结果

Compound	Contents/(mg/kg)			
	No. 1 (black)	No. 2 (brown)	No. 3 (coffee)	No. 4 (red)
Phenol	-	15	-	-
o-Phenylene-diamine	-	310	-	-
p-Phenylene-diamine	720	2120	627	890
2-Aminophenol	-	380	160	-
p-Aminophenol	453	1231	194	56
m-Aminophenol	324	534	617	436
p-Dihydroxybenzene	-	170	-	-
m-Dihydroxybenzene	120	310	-	-

-: not detected.

## 3 结论

本研究建立了一种同时检测22种染发剂成分的方法,该方法基于新型3D固定相联合超声辅助溶剂萃取-GC-MS测定染发剂中限用和禁用成分,具有良好的灵敏度和精密度,可为染发剂中限用和禁用成分含量的测定提供快速、高效、可靠的分析手段。结果表明,该方法萃取速度快、分离效果好,为染发剂中有害成分的检测提供了更好的技术支持。
